# Tetracyclines Removal from Aquaculture Effluents by Waste Mussel Shells After Thermal Modification

**DOI:** 10.3390/molecules31183219

**Published:** 2026-09-12

**Authors:** Hongmei Hu, Tongtong Zhang, Meiying Ye, Zhenhua Li, Tiejun Li, Yuanming Guo

**Affiliations:** 1 Key Laboratory of Sustainable Utilization of Technology Research for Fisheries Resources of Zhejiang Province, Zhejiang Marine Fisheries Research Institute, Zhoushan 316021, China; 2 Institute of Marine and Fisheries, Zhejiang Ocean University, Zhoushan 316021, China; 3 College of Material, Chemistry and Chemical Engineering, Key Laboratory of Organosilicon Chemistry and Material Technology, Ministry of Education, Hangzhou Normal University, Hangzhou 311121, China

**Keywords:** mussel shells-based material, *Mytilus coruscus*, tetracycline antibiotics, aquaculture effluents, removal

## Abstract

As a class of low-cost and broad-spectrum antibiotics in wide aquaculture use, residues of tetracyclines (TCs) are imperiling aquatic organisms and public health worldwide, leading to a high demand for TC removal from the aquaculture environment. In addition, considerable consumption of thick-shell mussels generates massive mussel shell waste, and their treatment has been an economic and environmental issue. Herein, a low-cost and high-efficiency mussel shells-based material was successfully prepared for TC removal by thermal modification of shell waste of *Mytilus coruscus*. Morphological and structural characterizations revealed the phase transformation of calcium carbonate to calcium oxide. The shell-based material calcined at 1050 °C (MC1050) yielded a Langmuir-fitted maximum removal capacity (q_m_) of 68.0 mg/g towards oxytetracycline (OTC). Thermodynamic calculations indicate that OTC removal by MC1050 is a spontaneous and endothermic process. OTC removal by MC1050 is likely achieved via multiple co-existing pathways, where Ca-OTC co-precipitation driven by CaO-phase hydration and resultant high-pH conditions dominates, accompanied by auxiliary surface-interaction contributions. Furthermore, MC1050 achieves removal efficiencies exceeding 96% for the ten target TCs spiked at 20 μg/L in real-world freshwater and marine aquaculture effluent matrices. Overall, the developed shell-based material shows promising performance for wastewater treatment and facilitates the resource utilization of waste mussel shells.

## 1. Introduction

The world aquaculture production is increasing rapidly to meet people’s growing demand for aquatic products. Among them, shellfish aquaculture accounts for 42.6% of global aquatic production [1] and has been regarded as a highly sustainable food source, which will be essential for global food security. However, their shells account for species dependent for 65–90% of live weight [2], being one of the most prominent wastes in the aquaculture industry. Every year, 10 million tons of waste oyster, clam, scallop and mussel shells are produced globally [2]. Most of them are directly landfilled or inappropriately released into the sea because of disposal costs as high as 150 USD/ton [3], resulting in waste of resources and severe damage to the coastal ecosystem. The European Union Directive has strongly enforced the development of new technologies to utilize shellfish waste as a sustainable resource [4]. Although shellfish waste is currently widely used in the fields of agriculture, medicine, construction, cosmetics, and food, the reutilization of shell wastes remains a great challenge in reducing environmental pollution and developing a circular economy.

Tetracycline antibiotics (TCs), including oxytetracycline (OTC), tetracycline (TC), chlortetracycline (CTC), and doxycycline (DXC), are widely applied in aquaculture to promote animal growth due to their low-cost and broad-spectrum antibacterial activity [5]. Due to their overuse and weak degradation ability, about 75% TCs are excreted in their active forms and persist in the environment. They accumulate along the food chain, cause toxicity to microbial communities enhance the evolution and spread of antibiotic resistance genes and disrupt the microbial flora in the human gut, posing a latent danger to aquatic organisms and public health worldwide. Much higher concentrations of TCs were found than sulfonamides in the coastal areas of Guangdong, China, with maximum values of 914 ng/L for OTC and 102 ng/L for CTC, which may be attributed to continuous wastewater discharge into the ocean by a nearby shrimp farm [6]. In typical marine aquaculture farms around Hailing Island, South China, OTC was found at the highest concentration of 15,163 ng/L, followed by TC (2305 ng/L) in the water samples of a shrimp larvae pond [7]. Furthermore, the predominant antibiotic from the discharged water in three aquaculture farms around the Bohai Bay was also OTC [8], while DXC was the dominant in typical aquaculture areas around the Yellow Sea with concentration of 9032 ng/L at the late aquaculture stage [9]. Therefore, effective elimination of TCs in aquaculture effluents is critical to human health and ecological safety.

Currently, the elimination of TCs from aquaculture effluents mainly relies on physical, chemical or biological methods, including adsorption [10], hydrolysis [11], photodegradation [12], advanced oxidation [13], biodegradation [14], electrochemical treatment [15], membrane filtration [16], constructed wetlands [17], and combined processes [18]. Among these techniques, adsorption has attracted considerable interest owing to its high efficiency, low-cost features, abundant material sources, and simple operation [10]. Nevertheless, practical application of adsorption requires high-performance and cost-effective sorbents. Beyond conventional sorbents such as activated carbon, zeolites and clay, materials derived from diverse natural feedstocks such as pine, poplar, and crop straws [10], crayfish shells and waste-activated sludge [19,20], rapeseed residue [21], algae (*Enteromorpha* and *Chlorella vulgaris*) [22], *Sargassum horneri* [23], wild almond shell [24], coconut shell and sawdust [25], rice husk and olive stones [26], etc., have gained growing attention. Such feedstocks are renewable and impose relatively low environmental burdens during service.

The thick-shell mussel *Mytilus coruscus* is one of the most prolific mussel species in Chinese aquaculture, especially those produced in Shengsi County, Zhoushan, which are known as the “Lady of the East China Sea” and are exported to Europe and the United States and other markets [27]. Despite economic benefits, the quantity of discarded mussel shells should not be underestimated since only 10–30% of mussel shell wastes have been recycled. The mussel shells are mainly composed of carbonate (CaCO_3_), and they can adsorb contaminants via precipitation, ion exchange, electrostatic interactions, etc. Recent studies have demonstrated the potential of thermally treated mussel shells for the removal of fluoride [28], Pb(II) [29], and mercury [30]. Nevertheless, most existing studies mainly focus on inorganic anions and heavy-metal ions, while investigations targeting tetracycline antibiotics remain limited. Since OTC is one of the most frequently used antibiotics in global aquaculture [6], we selected it as a model compound to study the TC removal potential by Shengsi *Mytilus coruscus* shell-based material. Rather than proposing an entirely new technical route, this work explores the feasibility of reusing waste mussel shells for elimination of TCs in both freshwater and marine aquaculture effluent matrices. This approach complies with circular economy principles. It provides scientific foundations and technical references for mitigating tetracycline-antibiotic pollution in mariculture and freshwater aquaculture environments, and offers insights into advancing the resource utilization of waste shells for sustainable aquaculture.

## 2. Results and Discussion

### 2.1. Characterization

**Figure 1:**
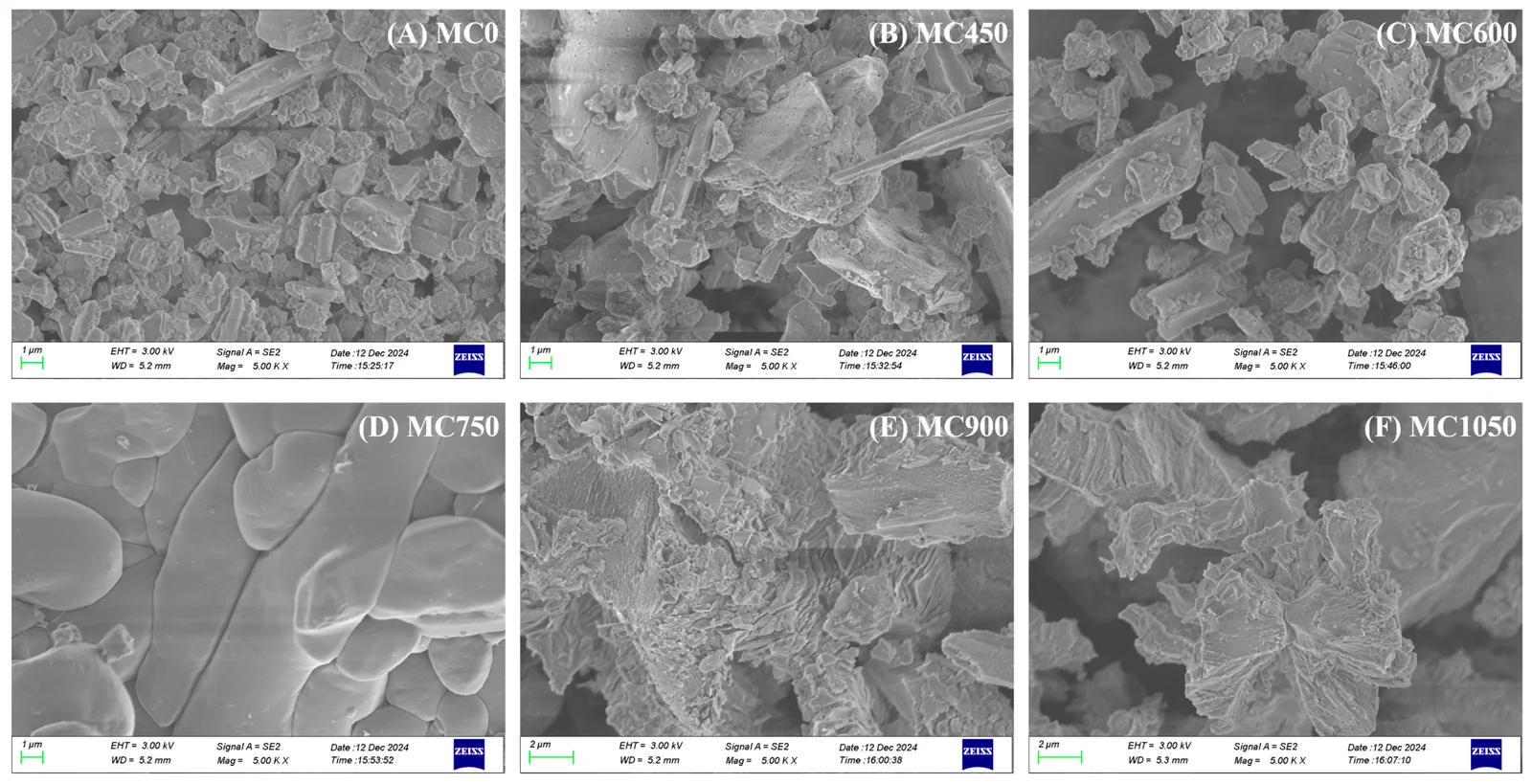
SEM images of the prepared *Mytilus coruscus* shell-based materials: (**A**) MC0 (raw shell powder), (**B**) MC450 (calcined at 450 °C), (**C**) MC600 (calcined at 600 °C), (**D**) MC750 (calcined at 750 °C), (**E**) MC900 (calcined at 900 °C), (**F**) MC1050 (calcined at 1050 °C).

**Figure 2:**
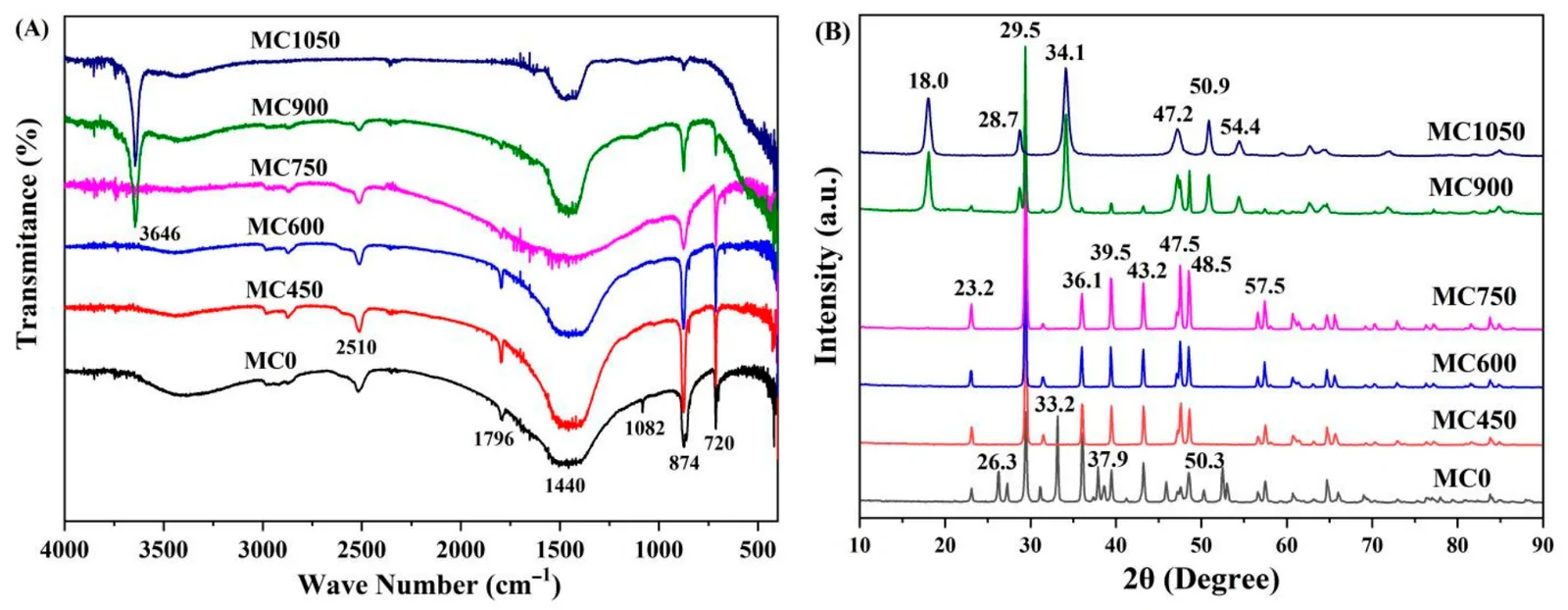
FTIR spectra (**A**) and XRD patterns (**B**) of the prepared *Mytilus coruscus* shell-based materials.

The pollutant-removal performance of a sorbent is generally associated with its microstructure. Scanning electron microscopy (SEM) enables observation of micro-morphological features of the as-prepared materials. As illustrated in Figure 1, raw MC0 is dominated by aragonite and calcite phases, which exhibit massive and granular morphologies with heterogeneous grain sizes [29,31]. This observation is consistent with the X-ray diffraction (XRD) results (Figure 2B). Following calcination, organic components were decomposed, and MC450 and MC600 developed looser structures with increased pore abundance. However, as the calcination temperature increased to 750 °C, the microstructure of MC750 changed remarkably; smooth and dense crystals aggregated into clusters, accompanied by melting and adhesion among crystal particles. By contrast, MC900 and MC1050 displayed distinct rough folded cracks. Compared with MC900, calcium-containing phases in MC1050 presented petal-shaped crystals, suggesting a more thorough decomposition of CaCO_3_.

Fourier-transform infrared spectroscopy (FTIR) and XRD analyses further verified the sintering phenomenon of calcium-oxide particles in the as-prepared shell-based materials (Figure 2). As displayed in Figure 2A, the bands at 720, 874, and 1440 cm^−1^ correspond to the in-plane bending vibration, symmetric stretching, and asymmetric stretching of CO_3_^2−^, respectively [32,33]. The peaks at 720 and 874 cm^−1^ are characteristics of the aragonite structure [29], whereas the peak at 1440 cm^−1^ is typical of calcite [29]. Furthermore, three weak peaks at 1082, 1796, and 2510 cm^−1^ originate from symmetric-stretching vibrations of calcite mixed with minor aragonite, C=O stretching vibrations, and O–C–O asymmetric stretching vibrations of CaCO_3_, respectively [34]. Accordingly, MC0 prior to calcination mainly consists of CaCO_3_, existing in the forms of calcite and aragonite. As the calcination temperature increased, multiple characteristic absorption bands assigned to CaCO_3_ (720, 874, 1082, 1796, and 2510 cm^−1^) gradually vanished. For MC900 and MC1050, a sharp absorption band emerged at 3646 cm^−1^, which is ascribed to the O-H stretching vibration of Ca(OH)_2_ [34]. This observation indicates that CaCO_3_ decomposes into CaO during high-temperature calcination, and the generated CaO tends to hydrate to form Ca(OH)_2_ upon exposure to moisture. Furthermore, the characteristic bands of CaCO_3_ at 720 cm^−1^ and 2510 cm^−1^ vanish completely for MC1050, while weak residual signals remain detectable for MC900, suggesting more thorough decomposition of calcium carbonate in MC1050 at an elevated calcination temperature. In aqueous environments, calcium ions released by MC900 and MC1050, together with surface hydroxyl sites, may form complexes and hydrogen bonds with functional groups of OTC (e.g., carboxyl, amino and hydroxyl groups), which could partially contribute to OTC removal [35]. The emergence of the –OH band at high calcination temperatures is consistent with the XRD findings.

The XRD patterns of MC0, MC450, MC600, MC750, MC900, and MC1050 are presented in Figure 2B. Raw MC0 is dominated by crystalline CaCO_3_, exhibiting intense diffraction peaks at 2θ = 29.5°, 36.1°, 39.5°, 43.2°, 48.5°, and 57.5°, which correspond to the calcite phase (PDF 83-1762), as well as peaks at 2θ = 26.3°, 33.2°, 37.9°, 50.3°, assigned to the aragonite phase (PDF 01-071-2396) [34]. As the calcination temperature increased from 450 °C to 750 °C, MC450, MC600, and MC750 displayed comparable diffraction profiles. The diffraction peaks belonging to the aragonite phase (2θ = 26.3°, 33.2°, 37.9°, and 50.3°) vanished, while the characteristic peaks of calcite gradually intensified, suggesting the phase transformation of aragonite to calcite prior to the thermal decomposition of CaCO_3_ [31]. With further temperature elevation, characteristic diffraction peaks of CaO at 2θ = 54.4° (PDF 00-037-1497) [33], together with peaks of Ca(OH)_2_ at 2θ = 18.0°, 28.7°, 34.1°, 47.2°, 50.9° (PDF 01-070-5492) [36], were detected for MC900 and MC1050, demonstrating the conversion of CaCO_3_ into CaO [29]. The formation of Ca(OH)_2_ originates from the reaction between CaO and atmospheric moisture [36]. Moreover, no characteristic calcite-phase peaks of CaCO_3_ were detected for MC1050, whereas residual calcite signals remained for MC900. This finding agrees with the FTIR results, implying that CaCO_3_-to-CaO conversion proceeded more completely in MC1050.

As summarized in Table 1, all as-prepared materials display pore sizes ranging from 4.22 to 17.61 nm, confirming their mesoporous structure. The molecular size of OTC (~0.79 nm) is considerably smaller than the material pore size, which implies that pore-filling could be structurally feasible for OTC removal [20]. The specific surface area and pore volume vary markedly with calcination temperature, showing a fluctuating trend. Among all tested samples, MC750 presents the lowest specific surface area and pore-volume values, while MC900 exhibits the highest porosity. At 750 °C, the phase transformation from aragonite into dense calcite is presumed to induce pore collapse and drastically reduce pore-structure parameters, as indirectly supported by BET and SEM observations (Figure S1). The elevated porosity of MC900 is speculated to stem from the formation and growth of CaO crystals upon carbonate decomposition.

Notably, MC1050 exhibits a drastically reduced specific surface area compared with MC900, which can be explained by the severe particle agglomeration under high calcination temperature. Such agglomeration collapses partial pore structure and decreases surface area, which is consistent with previous findings [37]. Importantly, although MC1050 has a much lower specific surface area, it achieves better OTC removal performance than MC900. This further indicates that physical pore structure and surface area are not the decisive factors for OTC removal. Instead, the superior surface chemical reactivity of MC1050 dominates the removal behavior.

### 2.2. Removal Performance

#### 2.2.1. Effect of Calcination Temperature

**Figure 3:**
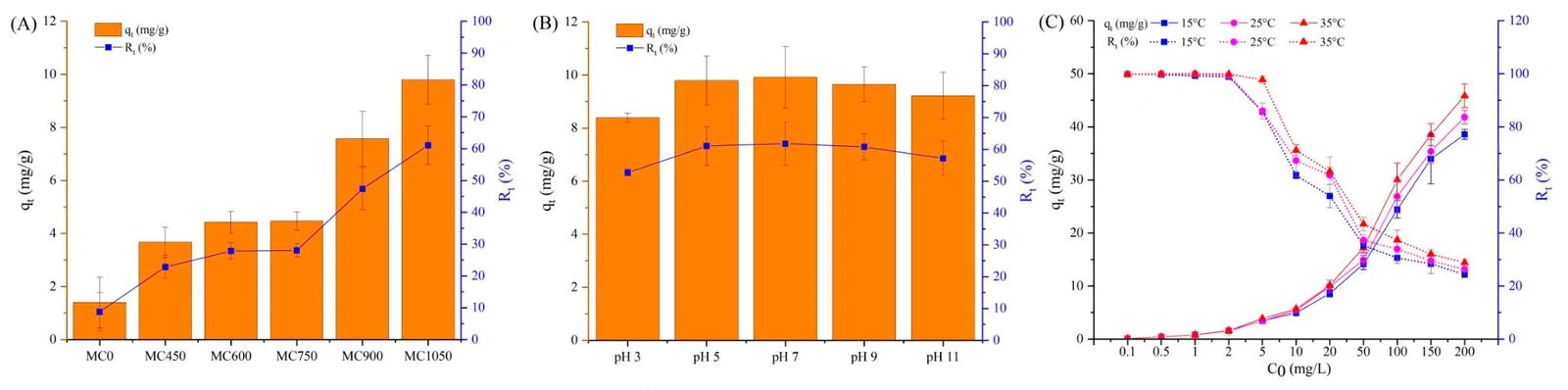
Effect of (**A**) calcination temperature, (**B**) initial solution pH, and (**C**) initial OTC concentration and equilibrium temperature on the removal capacity and removal rate.

Figure 3A presents the OTC-removal capacity of the as-prepared materials, showing a gradual increase from MC0 to MC750 and a sharp rise for MC900 and MC1050. ANOVA results indicate that the calcination temperature significantly affects OTC-removal performance (F = 46.492, *p* < 0.05). According to the characterization results in Section 2.1, samples obtained at low calcination temperatures consist mainly of CaCO_3_, and OTC removal is presumed to rely largely on physical pore-filling effects [20]. As the calcination temperature increases to 900 °C, CaO becomes the dominant phase. Accordingly, besides pore-filling, interfacial interactions including complexation and hydrogen-bonding exert more pronounced contributions to OTC removal [3,20]. FTIR and XRD analyses corroborate the superior OTC-removal performance of MC1050 relative to MC900. XRD patterns verify that MC1050 is dominated by crystalline CaO with high hydration activity. Upon exposure to water, CaO hydrates to form Ca(OH)_2_. It is speculated that in situ-formed Ca(OH)_2_ may participate in chemical-bonding interactions with OTC molecules and contribute to improved removal efficiency [28]. Nevertheless, direct experimental evidence supporting this proposed pathway remains limited. Accordingly, MC1050 was selected as the optimal material for subsequent experiments.

#### 2.2.2. Effect of Initial Solution pH

Solution pH determines the surface charge of solids and contaminant speciation, and thus plays an important role in pollutant removal performance [20,28]. Only the initial pH was adjusted in this experiment. Due to rapid hydration of CaO-related phases in MC1050, substantial OH^−^ is released, and the final pH of all systems stabilizes at 12.1–12.3 across the preset initial-pH range of 3–11. This demonstrates that the actual reaction environment is controlled by MC1050-derived alkalinity rather than the pre-adjusted initial pH.

As shown in Figure 3B, OTC removal capacity rises first and then declines with increasing initial pH (3–11), reaching the maximum capacity of 9.92 mg/g (removal efficiency 61.78%) at an initial pH of 7. The pK_a_ values of OTC are 3.27, 7.32 and 9.11, corresponding to cationic, zwitterionic and anionic forms [38]. Given the strongly alkaline final pH, OTC predominantly exists in anionic form at equilibrium. Dissolved Ca^2+^ released from material hydration may chelate anionic OTC to form Ca-OTC co-precipitates, contributing to OTC removal. At lower initial pH, H^+^ may consume hydroxide released from hydrated CaO-bearing phases in the early reaction stage. Such neutralization may influence Ca-OTC chelation and precipitation, lowering OTC removal capacity. As initial pH rises, less hydroxide is depleted by neutralization; sufficient Ca^2+^ may promote the formation of Ca-OTC precipitates and improve removal performance, reaching the maximum capacity at initial pH 7. Under high initial-pH conditions, excess OH^−^ may trigger Ca(OH)_2_ precipitation, which may shield reactive sites and consume free Ca^2+^ available for Ca-OTC complexation, thereby reducing removal efficiency.

Notably, OTC removal capacity of MC1050 only varies moderately (8.39–9.92 mg/g) over the tested initial-pH range of 3–11, indicating that this 1050 °C–calcined shell-based material delivers relatively stable removal performance under broadly variable initial-pH conditions. Even so, the material tends to alkalinize aqueous solutions, which means pH regulation may still be necessary for practical aquaculture wastewater applications.

Taken together, OTC removal by MC1050 likely proceeds via multiple coexisting pathways. Hydration of CaO-bearing phases releases abundant calcium species and elevates solution pH to highly alkaline levels. Under these high-calcium, strongly alkaline conditions, co-precipitation of Ca-OTC complexes is proposed as the dominant pathway, while transient interfacial surface complexation during material hydration serves as an auxiliary mechanism. Alkaline conditions may trigger mild OTC hydrolysis [39], potentially overestimating apparent removal efficiency; nevertheless, Ca^2+^ is reported to markedly suppress OTC hydrolysis, especially in alkaline solution [40]. Notably, the relative contribution of each pathway cannot be quantified in this work, because post-reaction solid characterization, zeta potential measurement, quantitative Ca^2+^ detection and mass balance analysis were not performed. 

#### 2.2.3. Effect of Initial OTC Concentration and Equilibrium Temperature

A major impetus is provided by the OTC initial concentration to conquer any opposition caused by OTC mass transfer between solid and liquid phases [41]. As exhibited in Figure 3C, the removal capacity was 0.08 mg/g at an initial OTC concentration of 0.1 mg/L under three equilibrium temperatures (15 °C, 25 °C, and 35 °C), with about a 100% removal rate. With increasing initial OTC concentration, OTC-removal capacity rises, owing to continuous occupation of available surface vacant sites. Nevertheless, removal efficiency gradually decreases once these sites become saturated [34]. Furthermore, the OTC-removal capacity of MC1050 increases gradually as temperature elevates from 15 °C to 35 °C, which can be attributed to intensified molecular motion at higher temperatures that raises the probability of OTC contacting reactive sites [29].

#### 2.2.4. Removal Isotherms and Thermodynamics

**Figure 4:**
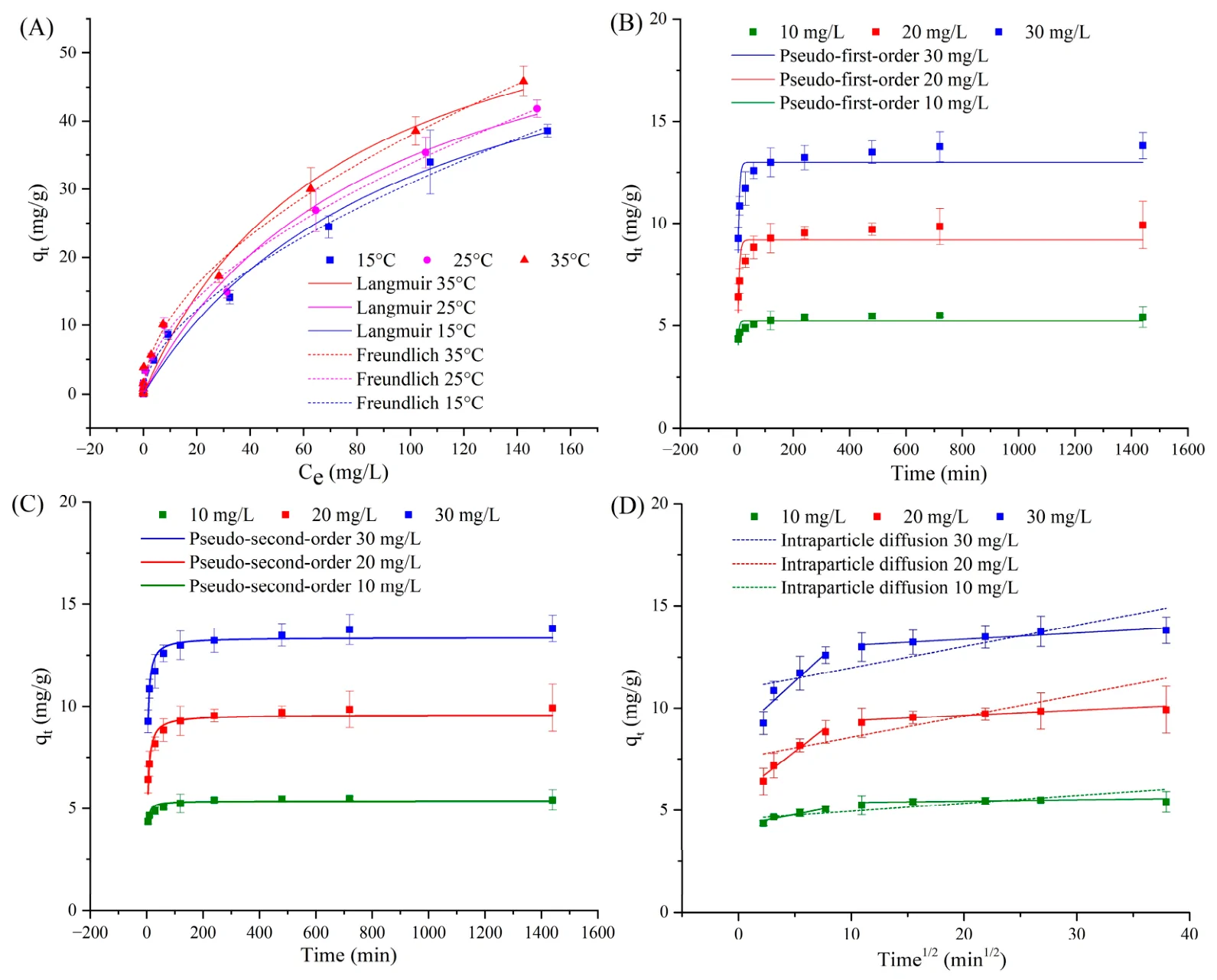
Curve fitting of (**A**) isothermal removal, (**B**) pseudo-first-order, (**C**) pseudo-second-order, and (**D**) intra-particle diffusion models.

Figure 4A displays the isotherm fitting curves for OTC removal by MC1050, and the corresponding fitted parameters are listed in Table 2. Across the three investigated temperatures, the Freundlich model produces slightly higher coefficients of determination (R^2^ > 0.99) compared with the Langmuir model (R^2^ > 0.97), which suggests heterogeneous interfacial properties during the OTC-removal process [20]. In addition, positive K_L_ values from the Langmuir model and Freundlich 1/n values lower than 1 indicate favorable interactions between OTC and the material [42,43]. Moreover, the Langmuir-predicated q_m_ for OTC removal by MC1050 rises with increasing temperature, reaching a maximum value of 68.0 mg/g at 35 °C. For reference, this fitted q_m_ value is higher than the reported values of KOH-modified cassava waste biochar (10.0 mg/g) [44], cauliflower leaves biochar (35.3 mg/g) [45], and lanthanum-modified zeolite (36.4 mg/g) [46], yet lower than modified chestnut-shell biochar (353 mg/g) [35] and *Enteromorpha prolifera* biochar (103 mg/g) [47] (Table S1). Note that these q_m_ data are obtained under divergent experimental conditions, so direct cross-study comparison has limitations.

This study further analyzed the relationship between lnK_C_ and 1/T (Figure S2), and thermodynamic parameters are list in Table 3. All ΔG^0^ values were negative and became more negative when temperature increased, suggesting that OTC removal onto MC1050 was spontaneous and became more favorable at higher temperatures [29,41,45,46,48]. Moreover, the positive ΔH^0^ value further verified the endothermic nature, and the positive ΔS^0^ value indicated the increasing randomness at the solid–liquid interface during OTC removal [29,41,45,46,48].

#### 2.2.5. Removal Kinetics

Fast removal kinetics and high removal capacity are desirable features for practical treatment materials [49]. As shown in Figure 4B, OTC-removal proceeds rapidly at the initial stage and gradually reaches equilibrium after approximately 120 min. This trend is attributed to abundant accessible reactive sites on MC1050 at the early stage, which are gradually consumed as the removal process proceeds [28,43,45,48]. Fitting curves from three classical kinetic models are displayed in Figure 4B–D, and corresponding fitted parameters are summarized in Table 4. Although the PSO model yields relatively higher R^2^ values (0.896–0.961) compared with the PFO model (R^2^ = 0.525–0.795), and its calculated q_e,cal_ values are closer to experimental q_e,exp_, high R^2^ and close q_e,cal_–q_e,exp_ agreement merely reflect satisfactory correlation between model outputs and experimental observations. While several previous studies have inferred chemisorption from PSO kinetic fitting [29,45,48,49], such kinetic outputs cannot provide conclusive evidence for better model performance or the occurrence of chemisorption in the present system. In short, mechanistic insights cannot be drawn exclusively from kinetic fitting results.

The IPD model shows two-stage linear segments and does not pass through the origin, suggesting that OTC removal follows a multi-step process [34,45,48,49]. The overall process may involve rapid surface-associated uptake of OTC, followed by slower intraparticle diffusion until dynamic equilibrium is established. The higher K_i1_ relative to K_i2_ demonstrates that the fast-stage surface-related process proceeds more rapidly than intraparticle diffusion within MC1050 [48].

#### 2.2.6. Effect of Ionic Strength and Humic Acid

As shown in Figure S3, the OTC-removal capacity of MC1050 exhibits a slight decline as the NaCl concentration increases from 0 to 5% (*m*/*v*). This observation implies that elevated ionic strength interferes with electrostatic interfacial interactions [34,42]. Nevertheless, the removal capacity decreases by only 13.5% even at the highest NaCl concentration, demonstrating that MC1050 retains considerable potential for OTC elimination in high-salinity aquaculture wastewater.

In addition, natural organic matter is widely present in actual water bodies. Hence, the influence of organic matter, represented by humic acid, on OTC removal, was investigated. As illustrated in Figure S4, low humic-acid concentrations (0–0.1 mg/L) slightly promote OTC removal. Abundant hydroxyl groups in humic acid can form complexes with OTC, which contributes to improved OTC elimination [20]. With further increases in humic-acid concentration, excess humic acid may block pore channels and compete with OTC for reactive sites on MC1050 [20], leading to reduced removal capacity. Even so, MC1050 still exhibits favorable OTC-removal performance at humic-acid concentrations below 3 mg/L.

### 2.3. Removal Application in Real-Water Matrices

To assess its practical applicability, three water matrices, including Wahaha pure water (pH 7.06), freshwater aquaculture effluent (pH 8.27), and marine aquaculture effluent (pH 8.44, salinity 18.9), were collected. Batch tests were performed on spiked water matrices (spiked with 0.8 mg/L OTC or 20 μg/L of ten TCs) to evaluate the TC-removal performance of 25 mg MC1050 at 25 °C. As shown in Table S2, removal efficiencies of the ten TCs reached 96.5–99.9% in the three 20 mL spiked water matrices (spiked at 20 μg/L for each TC), indicating favorable performance for TC removal in aquaculture water matrices. Furthermore, the OTC removal capacities of MC1050 in 500 mL spiked Wahaha pure water, spiked freshwater aquaculture effluent, and spiked marine aquaculture effluent were 8.61 ± 0.60 mg/g, 8.65 ± 1.98 mg/g, and 7.92 ± 0.87 mg/g, respectively. Such discrepancies may stem from differences in initial pH, ionic strength, and co-existing components among the three water samples. Notably, tetracycline residues in the collected aquaculture effluents were only at ng/L levels, and most target TCs were below detection limits. Accordingly, artificially fortified real-water samples were used in this work for valid performance evaluation. These results describe pollutant-removal behavior in aquaculture-relevant water matrices, rather than remediation of intrinsically contaminated field samples. Collectively, MC1050 exhibits promising potential for TC elimination in aquaculture-derived water systems.

Notably, the formed Ca-OTC precipitates may be attached to the solid phase, making conventional desorption and regeneration difficult. Thus, MC1050 functions as a disposable water-purifying material: spent solids can be separated by natural sedimentation and manual salvage for centralized harmless disposal rather than regeneration, preventing secondary antibiotic release. This non-regenerable feature my impose constraints on its economic performance and application scheme. Moreover, the residual calcium matrix can act similarly to quicklime to regulate water alkalinity and improve sediment quality. Nevertheless, systematic safety assessments are required to evaluate long-term residue accumulation risks before field application.

## 3. Materials and Methods

### 3.1. Chemicals and Reagents

*Mytilus coruscus* shells were collected from Shengsi, Zhoushan, China. OTC, 4-epioxytetracycline (EOTC), methacycline (MTC), TC, 4-epitetracycline (ETC), DXC, meclocycline (MCC), chlortetracycline (CTC), 4-epichlortetracycline (ECTC), and isochlortetracycline (ICTC) were supplied by Alta Scientific Co., Ltd. (Tianjin, China). OTC-^13^C_22_^15^N_2_ was obtained from Dr. Ehrenstorfer GmbH (Augsburg, Germany). TC-D_6_, DXC-D_3_, and CTC-^13^C-D_3_ were purchased from Toronto Research Chemicals (Toronto, ON, Canada). HPLC-grade methanol and formic acid were provided by Merck (Darmstadt, Germany), and humic acid was obtained from Alta Scientific Co., Ltd. (Tianjin, China). Sodium chloride (NaCl) and ethylenediaminetetraacetic acid disodium salt (Na_2_EDTA) were supplied by Sinopharm Chemical Reagent Co., Ltd. (Beijing, China). Ultrapure water was produced by a Milli-Q Plus 185 system (Millipore Corporation, Burlington, MA, USA).

### 3.2. Sampling and Preparation

Wahaha pure water samples (pH 7.06) were obtained from local shop stores, and freshwater aquaculture effluents (pH 8.27) and marine aquaculture effluents (pH 8.44, salinity 18.9) were collected from a freshwater fish farm in Linhai (121.2736° E and 28.7540° N) and a South American white shrimp factory farm in Sanmen (121.5644° E and 29.1608° N), Zhejiang Province, respectively. The collected aquaculture effluents were pressure filtered through 60 mm diameter glass fiber filters (0.45 μm pore size) to remove particulates and then stored at 3 °C until removal experiments.

### 3.3. Preparation of the Thick-Shell Mussel-Based Materials

**Figure 5:**
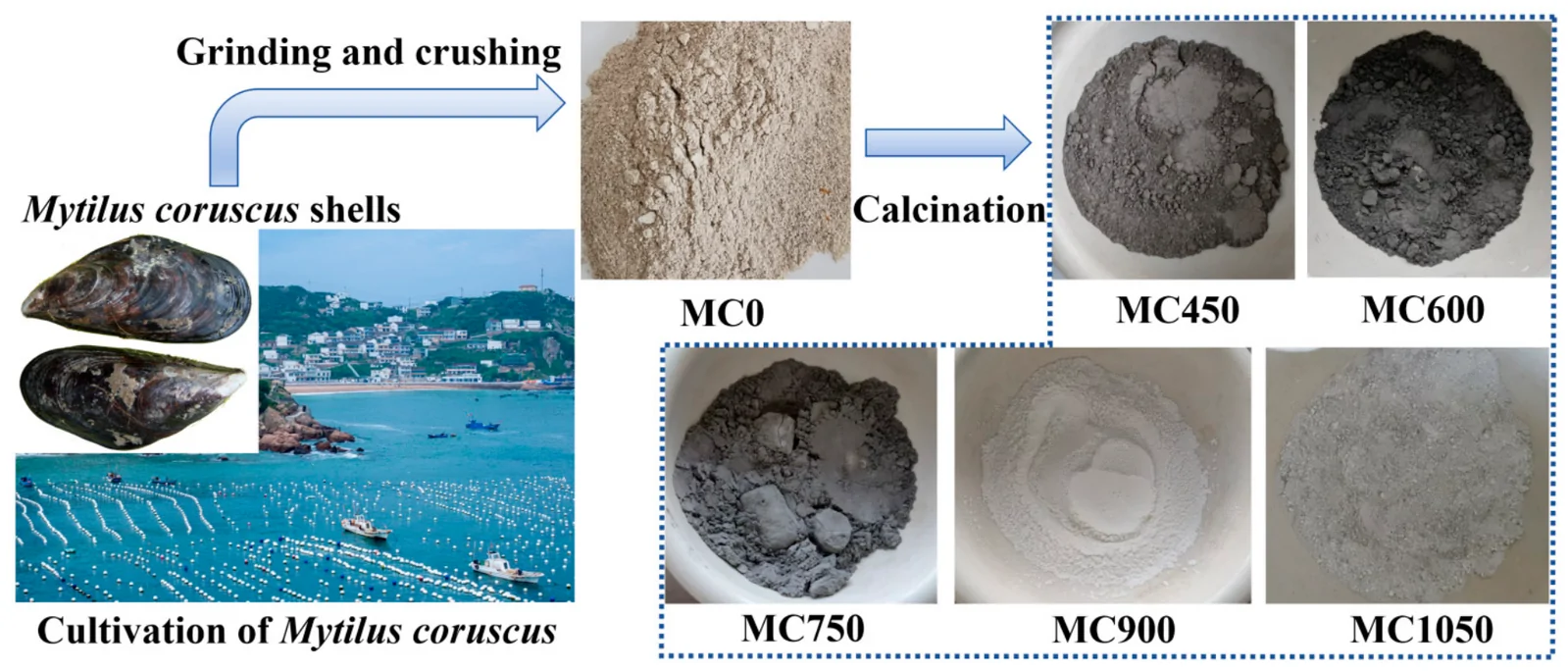
Preparation of *Mytilus coruscus* shell-based materials (MC0 to MC1050) by thermal modification.

The schematic diagram of the preparation process is shown in Figure 5. Briefly, the waste *Mytilus coruscus* shells were collected and thoroughly cleaned to remove residual flesh and impurities. The cleaned shells were oven-dried at 80 °C for 48 h. After drying, the shell samples were ground and crushed using a disk vibration grinder (Retsch RS200, RETSCH GmbH, Haan, Germany) and sieved through a 160-mesh screen to obtain homogeneous fine powder (denoted as MC0). The prepared MC0 was further calcined in a tube furnace (OTF-1200X-50, Hefei Kejing Materials Technology Co., Ltd., Hefei, China) under a gentle nitrogen flow to maintain an inert atmosphere. The heating procedure was set as follows: the furnace was first heated from room temperature to 150 °C at a rate of 10 °C/min and maintained for 2 h; subsequently, the temperature was continuously increased to the target calcination temperatures (450 °C, 600 °C, 750 °C, 900 °C, and 1050 °C) at 5 °C/min and held for 2 h. The final calcined materials were denoted as MC450, MC600, MC750, MC900, and MC1050, respectively. To avoid spontaneous hydration and carbonation of active CaO components caused by exposure to ambient air, all samples were immediately stored in a desiccator prior to characterization and removal experiments.

### 3.4. Characterization of the Shell-Based Materials

Surface morphologies of the as-prepared materials were characterized via field emission scanning electron microscopy (FE-SEM, Sigma 500, Carl Zeiss AG, Oberkochen, Germany). Brunauer–Emmett–Teller (BET) specific surface areas and porosity measurements were performed using a Micromeritics analyzer (ASAP 2460, Micromeritics, Norcross, GA, USA). Functional groups on the materials surface were analyzed by Fourier-transform infrared spectroscopy (FTIR, Nicolet iS5, ThermoFisher, Waltham, MA, USA). The crystallinity and phase compositions were identified by X-ray diffraction (XRD, Dmax 2550, Rigaku, Akishima, Tokyo, Japan).

### 3.5. Removal Experiment

Batch removal experiments were performed in triplicate to explore the effects of various experimental parameters (i.e., calcination temperature, initial solution pH, initial OTC concentration, equilibrium temperature, contact time, NaCl concentration, and humic acid concentration), with OTC selected as the model compound. Table S3 lists the experiment design, one variable at each run. Each flask was loaded with 25 mg of the as-prepared materials and 20 mL of OTC aqueous solution, and incubated in a large-capacity full-temperature oscillator (DHZ-D, Suzhou Peiying Experimental Equipment Co., LTD, Suzhou, China) at 100 rpm in the dark. To assess its practical applicability, collected Wahaha pure water and natural aquaculture effluents were spiked with either 800 μg/L OTC or 20 μg/L of 10 TCs, which were employed to verify the TC-removal performance of the as-prepared shell-based materials. Concentrations of OTC and ten target TCs before and after the removal were quantified using a Waters ACQUITY UPLC I-Class system coupled to a Xevo TQ-S triple quadrupole mass spectrometer (UPLC-MS/MS, Waters, Milford, MA, USA). Analyses were performed under positive electrospray ionization (ESI+) with the multiple reaction monitoring (MRM) mode, according to our previous work [38]. MRM parameters for the target TCs are summarized in Table S4. After removal, all aqueous samples were centrifuged and then filtered. Depending on approximate analyte concentrations, the filtered samples were appropriately diluted or concentrated to match the instrument’s valid detection range. An aliquot of the resulting sample was added with Na_2_EDTA at 0.5 g/L (adjusted to pH 3), spiked with 20 ng of each isotope-labeled internal standard (ILIS), and then subjected to solid-phase extraction using CNW Poly-Sery HLB cartridges (500 mg, 6 mL, CNW Technologies, Duesseldorf, Germany) [38].

### 3.6. Data Analysis

The removal capacity (q_t_, mg/g) and removal rate (R_t_, %) at time t were calculated using Equations (1) and (2), respectively.

(1) $$q_{t}=\frac{\left(C_{0}-C_{t}\right)}{W}$$

(2) $$R_{t}=\frac{\left(C_{0}-C_{t}\right)}{C_{0}}\times 100\%$$

where C_0_ and C_t_ are the initial and residual concentration of OTC (mg/L), respectively. V and W stand for solution volume (L) and shell-based material dosage (g), respectively.

To clarify the removal behaviors and underlying mechanisms, three classic kinetic models including pseudo-first-order (PFO) [48], pseudo-second-order (PSO) [48], and intra-particle diffusion (IPD) [48], and two well-known isotherm models like the Langmuir [41] and the Freundlich [41] were applied. The detailed equations and corresponding parameters for these models are provided in Table S5.

Furthermore, OTC removal thermodynamics for the as-prepared shell-based material were examined at three distinct temperatures. The thermodynamics parameters, namely the standard Gibbs free energy change (ΔG^0^, kJ/mol), standard enthalpy change (ΔH^0^, kJ/mol)), and standard entropy change (ΔS^0^, J/mol/K), were derived according to Equations (3)–(5) [50]:

(3) ΔG^0^ = −RTlnK_C_

(4) ΔG^0^ = ΔH^0^ − TΔS^0^

(5) K_C_ = M_W_ × 55.5 × 1000 × K_L_

where K_C_ denotes the equilibrium constant, K_L_ is the Langmuir constant (L/mg), and factor 55.5 corresponds to the molar amount of pure water per liter. M_W_ represents the molecular weight of the pollutant (g/mol), with a value of 460.44 g/mol for OTC. The term M_W_ × 55.5 × 1000 × K_L_ is dimensionless. R stands for the universal gas constant (8.314 J/mol/K), and T is the absolute temperature in Kelvin (K).

### 3.7. Quality Assurance and Quality Control

The internal standard curve of each analyte (0.05–100 μg/L) was used for quantification (R^2^ > 0.99). For each batch of samples, procedural blanks, ILIS for each sample, matrix spikes, and sample duplicates were analyzed for quality control. No target TCs were detected in procedural blanks. Instrumental limits of detection (LODs) ranged from 0.03 to 0.10 μg/L (S/N = 3, Table S6). Analytes yielding concentrations below the instrumental LOD were treated as below-LOD values, and one-half of the corresponding LOD was applied for subsequent statistical calculations. Recoveries of target TCs in Wahaha pure water, freshwater aquaculture effluent, and marine aquaculture effluent were in the ranges of 82–116%, 84–120%, and 84–121%, respectively, with relative standard deviations (RSDs) of 4.6–8.7%, 0.5–9.8%, and 1.5–5.9% (*n* = 3) (Table S6).

### 3.8. Statistical Analysis

Quantitative results are expressed as mean ± standard deviation (SD). Statistical analysis was conducted using Origin 2024b (version 10.1.5.132). One-way ANOVA was applied to evaluate the statistical significance of calcination-temperature effects, followed by Tukey’s post hoc multiple comparison test with a significance level of *p* < 0.05.

## 4. Conclusions

In summary, a highly efficient shell-based material for TC removal was successfully fabricated via thermal modification of waste *Mytilus coruscus* shells. Characterization results verify that the major phase transforms from CaCO_3_ to CaO upon calcination. MC1050, the material calcined at 1050 °C, yielded a Langmuir-fitted maximum removal capacity (q_m_) of 68.0 mg/g toward OTC. Kinetics and isotherms outcomes showed that the Freundlich, PSO, and IPD models offered superior mathematical fits to describe the removal behavior. Thermodynamics analyses suggested that the OTC-removal process was endothermic and spontaneous. OTC elimination by MC1050 is probably accomplished through multiple co-existing pathways. Ca-OTC co-precipitation, driven by hydration of CaO-bearing phases and the consequent high-pH microenvironment, may constitute the dominant removal pathway, whereas surface-mediated interactions may contribute in an auxiliary capacity. Moreover, MC1050 achieves satisfactory TC-removal performance in spiked real-world freshwater and mariculture effluent matrices. The reuse of readily accessible waste mussel-shell resources provides an alternative strategy for eliminating tetracycline-antibiotic residues from aquaculture wastewaters. Nevertheless, high-temperature calcination for material preparation involves energy-associated environmental trade-offs. Accordingly, future efforts will focus on lowering the energy cost of material fabrication, evaluating long-term ecological risks under field-relevant conditions, and advancing the practical application of this shell-based material for real aquaculture-wastewater treatment.

## Figures and Tables

**Table 1:** Specific surface area, pore volume, and pore size of the prepared materials.

Material	Specific Surface Area (m^2^/g)	Pore Volume (cm^3^/g)	Pore Diameter (nm)
MC0	1.91	0.0084	17.61
MC450	2.96	0.0106	14.36
MC600	3.27	0.0104	12.70
MC750	1.39	0.0015	4.22
MC900	6.78	0.0176	10.40
MC1050	2.99	0.0083	11.16

**Table 2:** Langmuir and Freundlich isotherm parameters at different equilibrium temperatures.

Equilibrium Temperature (K)	Langmuir			Freundich		
	q_m_ (mg/g)	K_L_ (L/mg)	R^2^	K_F_ ((mg/g)(L/mg)^1/n^)	1/n	R^2^
308.15	68.0	0.0134	0.9804	3.19	0.5373	0.9935
298.15	65.8	0.0112	0.9758	2.67	0.5504	0.9922
288.15	64.0	0.0099	0.9803	2.18	0.5752	0.9924

**Table 3:** Thermodynamic parameters for OTC removal by MC1050.

Equilibrium Temperature (K)	lnK_C_	ΔG^0^ (kJ/mol)	ΔH^0^ (kJ/mol)	ΔS^0^ (J/mol/K)
308.15	12.74	−32.65	11.04	141.66
298.15	12.56	−31.15		
288.15	12.44	−29.80		

**Table 4:** The kinetic parameters for OTC removal by MC1050 at different initial concentrations.

C_0_	Pseudo-First-Order Model			Pseudo-Second-Order			Intra-Particle Diffusion Model (Segmented)						Intra-Particle Diffusion Model (Overall)		
(mg/L)	k_1_	q_e,cal_	R^2^	k_2_	q_e,cal_	R^2^	k_i1_	C_1_	R_1_^2^	k_i2_	C_2_	R_2_^2^	k_i_	C	R^2^
	(1/min)	(mg/g)		(g/mg/min)	(mg/g)		(mg/g/min^1/2^)	(mg/g)		(mg/g/min^1/2^)	(mg/g)		(mg/g/min^1/2^)	(mg/g)	
10	0.3016	5.21	0.5275	0.1374	5.36	0.8963	0.1090	4.24	0.8372	0.0067	5.30	0.4856	0.0385	4.56	0.7927
20	0.1900	9.19	0.5151	0.0307	9.59	0.9057	0.4265	5.73	0.9328	0.0254	9.13	0.8315	0.1039	7.53	0.6964
30	0.2160	12.99	0.7950	0.0112	13.37	0.9613	0.5017	8.79	0.8432	0.0304	12.77	0.8464	0.1045	10.92	0.5473

## Data Availability

The original contributions presented in this study are included in the article and Supplementary Materials. Further inquiries can be directed to the corresponding authors.

## Supplementary Materials

The following supporting information can be downloaded at: https://www.mdpi.com/article/10.3390/molecules31183219/s1, Figure S1: SEM image of MC750 at 10,000× magnification; Figure S2: Plot of lnK_C_ versus 1/T used to calculate thermodynamic parameters for OTC removal by MC1050; Figure S3: Effect of the NaCl concentration on OTC removal performance; Figure S4: Effect of the humic acid concentration on OTC removal performance; Table S1: Comparison of the OTC removal capacity of MC1050 and other materials; Table S2: TC-removal performance of MC1050 in three spiked real-world water samples; Table S3: Batch removal experiments designed for OTC; Table S4: Properties and MRM acquisition parameters for the target TCs based on our previous work [38]; Table S5: The kinetics and isotherm models used for OTC removal by the prepared shells-based materical; Table S6: The instrumental LODs and recoveries of 10 TCs in Wahaha pure water, freshwater aquaculture effluent and marine aquaculture effluent.
