# Supplementary material for: Tetracyclines Removal from Aquaculture Effluents by Waste Mussel Shells After Thermal Modification
Source: Molecules. 2026 Sep 12;31(18):3219. doi: 10.3390/molecules31183219 (PMC13609504; doi:10.3390/molecules31183219)
Supplement: Supplementary file 1 [file molecules-31-03219-s001.zip › molecules-4541714-supplementary.pdf]

# SUPPORTING INFORMATION

**Tetracyclines removal from aquaculture effluents by waste mussel shells after thermal modification**

Hongmei Hu[1,2], Tongtong Zhang[1,2], Meiying Ye[3], Zhenhua Li[1,2,*], Tiejun Li[1,2,*] and Yuanming Guo[1,2]

[1] Key Laboratory of Sustainable Utilization of Technology Research for Fisheries Resources of Zhejiang Province, Zhejiang Marine Fisheries Research Institute, Zhoushan 316021, China.

[2] Institute of Marine and Fisheries, Zhejiang Ocean University, Zhoushan 316021, China.

[3] College of Material, Chemistry and Chemical Engineering, Key Laboratory of Organosilicon Chemistry and Material Technology, Ministry of Education, Hangzhou Normal University, Hangzhou 311121, China.

Corresponding Author

Zhenhua Li

* Phone: +86-580-2299833; Fax: +86-580-2299881; E-mail: lzh0580@zjou.edu.cn

Tiejun Li

* Phone: +86-580-2299886; Fax: +86-580-2299881; E-mail: litiejun@zjou.edu.cn

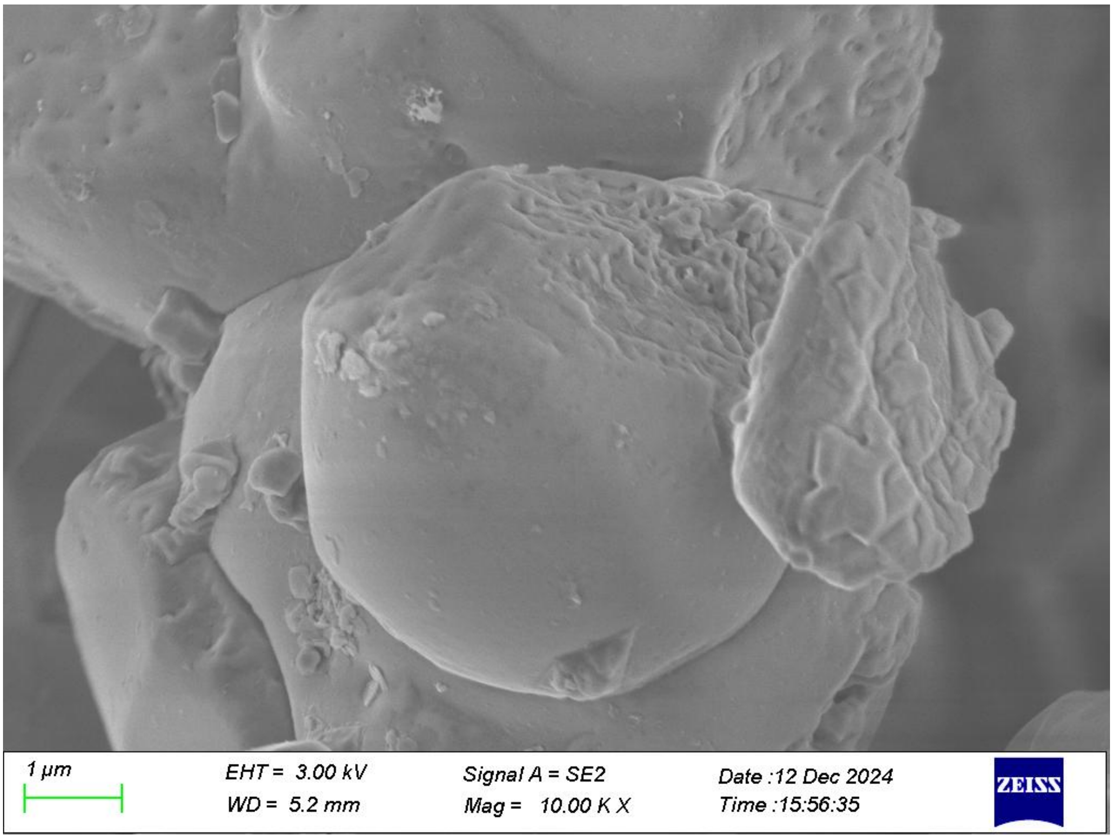


**Figure S1. SEM image of MC750 at 10 000 × magnification.**

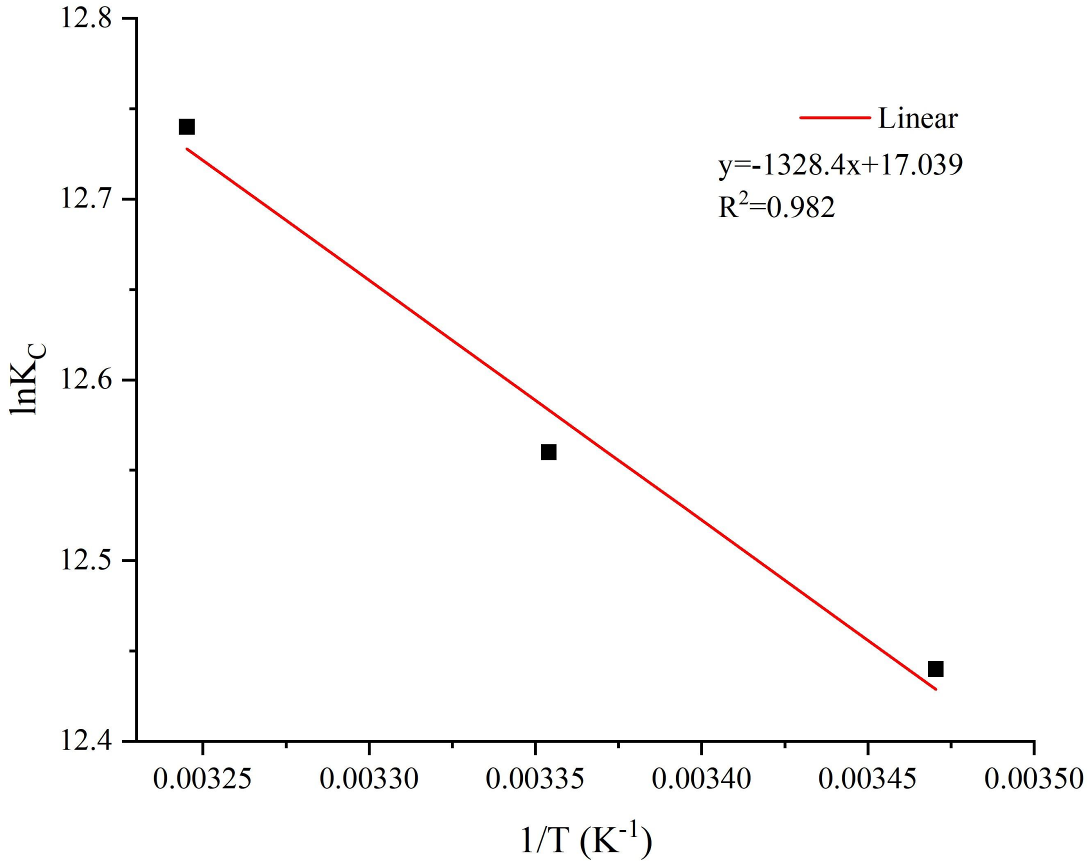


**Figure S2. Plot of $lnK_C$ versus 1/T used to calculate thermodynamic parameters for OTC removal by MC1050.**

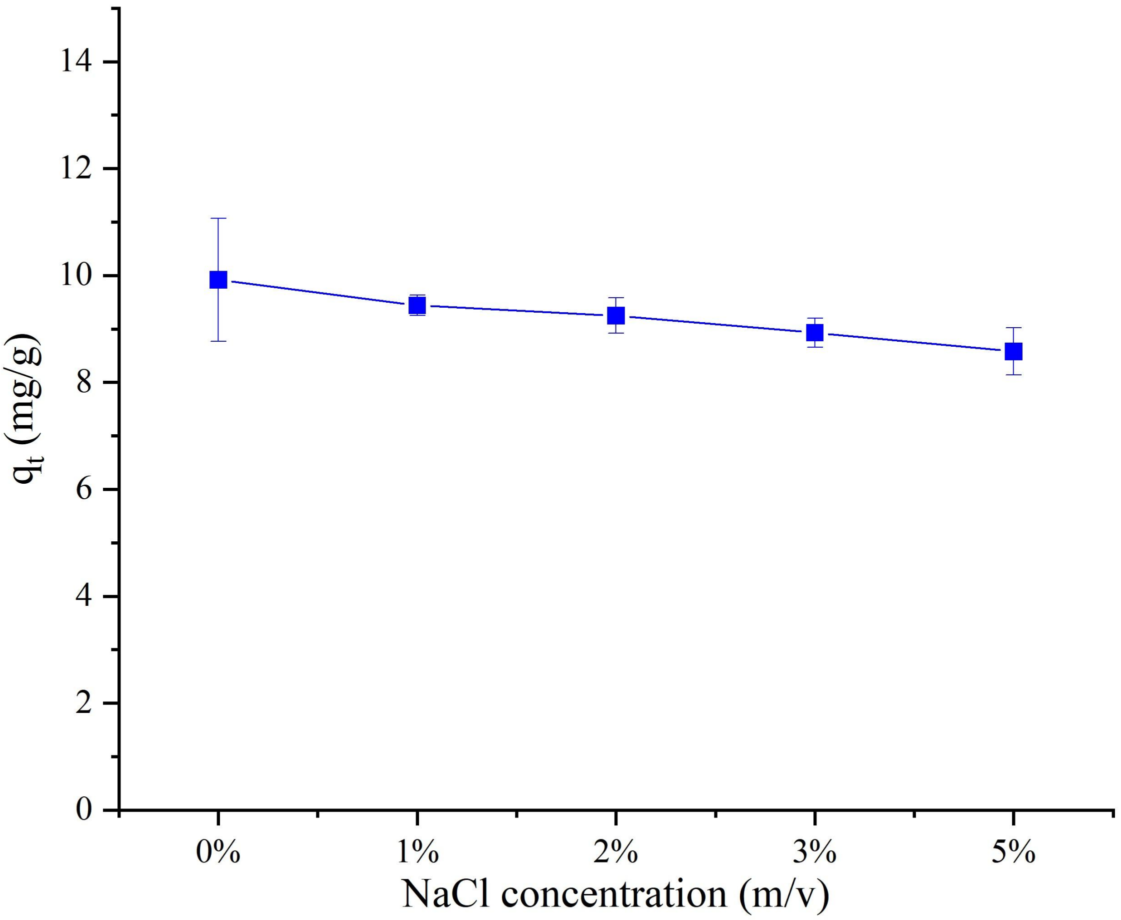


**Figure S3. Effect of the NaCl concentration on OTC removal performance.**

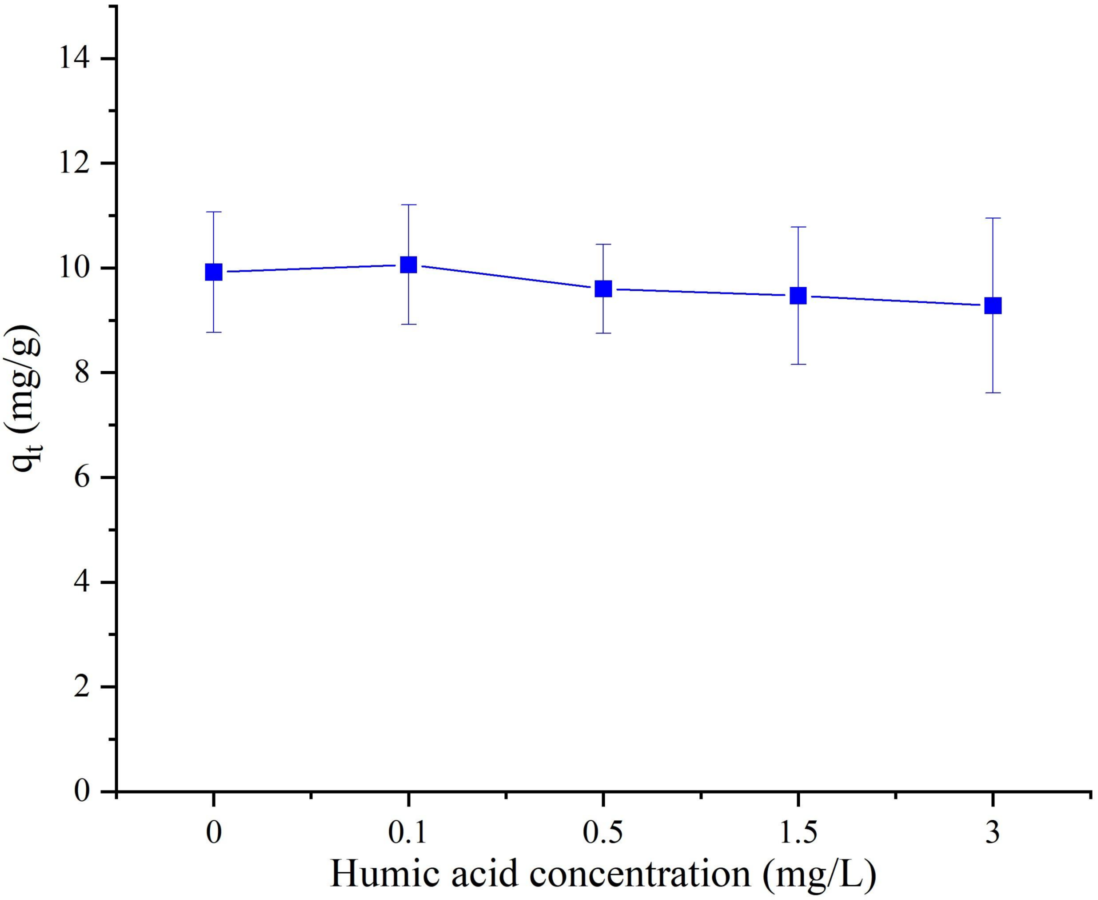


**Figure S4. Effect of the humic acid concentration on OTC removal performance.**

**Table S1. Comparison of the OTC removal capacity of MC1050 and other materials.**

| Materials | Raw material | Equilibrium temperature (K) | Material dosage (mg) | OTC initial concentration range (mg/L) | Contact time (min) | Langumir-fitted $q_m$ (mg/g) | Reference |
|---|---|---|---|---|---|---|---|
| Modified biochar derived from chestnut shell | chestnut shell | 318.15 | 50 | 20–400 | 600 | 353 | [35] |
| KOH-modified cassava waste biochar | cassava waste | 298.15 | 100 | 5–50 | 2880 | 10.0 | [44] |
| Cauliflower leaves biochar | cauliflower leaves | 308.15 | 50 | 10–150 | 2160 | 37.2 | [45] |
| *Enteromorpha prolifera* biochar | *Enteromorpha prolifera* | 298.15 | 10 | 60–150 | 1440 | 103 | [47] |
| Lanthanum modified zeolite | zeolite | 318.15 | 100 | 0–300 | 20 | 36.4 | [46] |
| MC1050 | *Mytilus coruscus* shells | 308.15 | 25 | 0.1–200 | 1440 | 68.0 | Present work |

**Table S2. TC-removal performance of MC1050 in three spiked real-world water samples.**

| TCs | Wahaha pure water (n=3) | | | Freshwater aquaculture effluent (n=3) | | | Marine aquaculture effluent (n=3) | | |
|---|---|---|---|---|---|---|---|---|---|
| | Background value[a] (ng/L) | Spiked: 20 µg/L of 10 TCs | Spiked:0.8 mg/L of OTC | Background value[a] (ng/L) | Spiked: 20 µg/L of 10 TCs | Spiked:0.8 mg/L of OTC | Background value[a] (ng/L) | Spiked: 20 µg/L of 10 TCs | Spiked:0.8 mg/L of OTC |
| | | Removal rate (%) | Removal capacity (mg/g) | | Removal rate (%) | Removal capacity (mg/g) | | Removal rate (%) | Removal capacity (mg/g) |
| OTC | <LOD | 99.3 | 8.61 ± 0.60 | 0.256 | 99.0 | 8.65 ± 1.98 | 47.3 | 98.3 | 7.92 ± 0.87 |
| EOTC | <LOD | 99.2 | / | <LOD | 98.4 | / | 7.32 | 99.6 | / |
| MTC | <LOD | 99.3 | / | <LOD | 99.6 | / | <LOD | 99.2 | / |
| TC | <LOD | 99.5 | / | 0.347 | 99.5 | / | 1.15 | 99.1 | / |
| ETC | <LOD | 99.6 | / | 0.083 | 99.5 | / | 0.738 | 99.6 | / |
| DXC | <LOD | 99.8 | / | 0.542 | 98.3 | / | <LOD | 98.1 | / |
| MCC | <LOD | 98.0 | / | <LOD | 96.6 | / | <LOD | 96.5 | / |
| CTC | <LOD | 98.8 | / | <LOD | 97.4 | / | <LOD | 98.1 | / |
| ECTC | <LOD | 99.5 | / | <LOD | 99.5 | / | <LOD | 99.6 | / |
| ICTC | <LOD | 99.9 | / | <LOD | 99.9 | / | <LOD | 99.5 | / |

a, Actual samples were concentrated 1000-fold by automated solid-phase extraction with CNW Poly-Sery HLB cartridges according our previous work[38].

**Table S3. Batch removal experiments designed for OTC.**

| | Experiment | Calcination temperature (°C) | Initial solution pH | Initial OTC concentration ($C_0$, mg/L) | equilibrium temperature (°C) | Contact time (min) | NaCl concentration (%, m/V) | Humic acid concentration (mg/L) |
|---|---|---|---|---|---|---|---|---|
| 1 | Effect of calcination temperature | Uncalcined and 450–1050 | 5 | 20 | 25 | 1440 | 0 | 0 |
| 2 | Effect of initial solution pH | 1050 | 3–11 | 20 | 25 | 1440 | 0 | 0 |
| 3 | Effect of initial OTC concentration and equilibrium temperature | 1050 | 7 | 0.1–200 | 15、25、35 | 1440 | 0 | 0 |
| 4 | Effect of contact time | 1050 | 7 | 10、20、30 | 25 | 5–1440 | 0 | 0 |
| 5 | Effect of the NaCl concentration | 1050 | 7 | 20 | 25 | 1440 | 0–5 | 0 |
| 6 | Effect of the humic acid concentration | 1050 | 7 | 20 | 25 | 1440 | 0 | 0–3 |

**Table S4. Properties and MRM acquisition parameters for the target TCs based on our previous work [38].**

| TCs | Molecular weight | Precursor ion (*m/z*) | Product ion (*m/z*) | Cone voltage (V) | Collision energy (eV) | Internal standard |
|---|---|---|---|---|---|---|
| Oxytetracycline (OTC) | 460.44 | 461.1 | 426.0*/442.9 | 20 | 20/15 | OTC-$^{13}C_{22}{}^{15}N_2$ |
| 4-Epioxytetracycline (EOTC) | 460.43 | 461.1 | 426.0*/442.9 | 20 | 20/15 | OTC-$^{13}C_{22}{}^{15}N_2$ |
| methacycline (MTC) | 442.42 | 443.1 | 380.9/426.3* | 34 | 20/25 | TC-$D_6$ |
| Tetracycline (TC) | 444.43 | 445.1 | 410.0*/427.0 | 25 | 20/14 | TC-$D_6$ |
| 4-epitetracycline (ETC) | 444.44 | 445.1 | 410.0*/427.0 | 25 | 20/14 | TC-$D_6$ |
| Doxycycline (DXC) | 444.44 | 445.2 | 410.0/428.0* | 30 | 24/18 | DXC-$D_3$ |
| Meclocycline (MCC) | 476.86 | 477.1 | 235.0/460.1* | 30 | 20/17 | CTC-$^{13}C$-$D_3$ |
| Chlortetracycline (CTC) | 478.88 | 479.1 | 444.1*/462.0 | 15 | 20/18 | CTC-$^{13}C$-$D_3$ |
| 4-Epichlortetracycline (ECTC) | 478.88 | 479.1 | 444.1*/462.0 | 15 | 20/18 | CTC-$^{13}C$-$D_3$ |
| Isochlortetracycline (ICTC) | 478.88 | 479.1 | 197.0/462.1* | 30 | 25/18 | CTC-$^{13}C$-$D_3$ |
| OTC-$^{13}C_{22}{}^{15}N_2$ | 484.44 | 485.1 | 449.0* | 20 | 20 | / |
| TC-$D_6$ | 450.47 | 451.1 | 416.0* | 25 | 20 | / |
| DXC-$D_3$ | 447.48 | 448.2 | 431.0* | 30 | 18 | / |
| CTC-$^{13}C$-$D_3$ | 482.88 | 483.1 | 448.1* | 15 | 20 | / |

* quantitative ion.

**Table S5. The kinetics and isotherm models used for OTC removal by the prepared shells-based materical.**

| Model | Equation | Symbol meaning | References |
|---|---|---|---|
| Pseudo-first order (PFO) | $q_t = q_e(1-e^{-k_1 t})$ | $k_1$ is the pseudo-first-order rate constant (1/min)<br>$q_t$ is the OTC removal amount at time t (mg/g) | [48] |
| Pseudo-second order (PSO) | $q_t = \frac{k_2 q_e{}^2 t}{(1+k_2 q_e t)}$ | $k_2$ is the pseudo-second-order rate constant (g/mg/min) | [48] |
| Intra-particle diffusion (IPD) | $q_t = k_{id} t^{\frac{1}{2}} + C_i$ | $C_i$ is the boundary layer thickness (mg/g)<br>$k_{id}$ is that diffusion rate constant (mg/g/min$^{1/2}$) | [48] |
| Langmuir | $q_e = \frac{q_m K_L C_e}{1+K_L C_e}$ | $K_L$ is the Langmuir constant (L/mg)<br>$q_m$ is the maximum removal capacity (mg/g) | [41] |
| Freundlich | $q_e = K_F C_e{}^{1/n}$ | $q_e$ is the removal capacity at equilibrium (mg/g)<br>$C_e$ is the concentration at equilibrium (mg/L)<br>$K_F$ is the Freundlich constant (mg/g)(L/mg)$^{1/n}$<br>$n$ is the exponential factor | [41] |

**Table S6. The instrumental LODs and recoveries of 10 TCs in Wahaha pure water, freshwater aquaculture effluent and marine aquaculture effluent.**

| TCs | Instrumental LOD (μg/L) | Wahaha pure water (n=3) | Freshwater aquaculture effluent (n=3) | Marine aquaculture effluent (n=3) |
|---|---|---|---|---|
| | | Spiked: 20 μg/L | Spiked: 20 μg/L | Spiked: 20 μg/L |
| | | Recovery (mean ± SD, %) | Recovery (mean ± SD, %) | Recovery (mean ± SD, %) |
| OTC | 0.03 | 102±8 | 98±10 | 101±6 |
| EOTC | 0.03 | 95±6 | 96±2 | 100±1 |
| MTC | 0.10 | 82±6 | 84±0.4 | 84±2 |
| TC | 0.03 | 91±6 | 95±1 | 95±2 |
| ETC | 0.03 | 108±9 | 114±2 | 113±3 |
| DXC | 0.03 | 111±9 | 117±2 | 115±3 |
| MCC | 0.06 | 103±7 | 109±2 | 107±4 |
| CTC | 0.03 | 94±7 | 103±1 | 100±3 |
| ECTC | 0.03 | 116±5 | 120±4 | 121±2 |
| ICTC | 0.03 | 83±4 | 93±2 | 89±2 |
